# An unexpected cardiac mass related to immunoglobulin G4-related disease

**DOI:** 10.1016/j.xjon.2026.101818

**Published:** 2026-04-16

**Authors:** Paul Dalmas, Paul Habert, Brigitte Vaunois, Nicolas Schleinitz

**Affiliations:** a Internal Medicine Department, Aix Marseille University, APHM, La Timone Hospital, Marseille, France; b Department of Radiology, Hôpital Nord, APHM, Marseille, France; c Aix Marseille University, LIIE, Marseille, France; d Department of Pathology, Medipath Aix-Marseille, Eguilles, France

**Figure:**
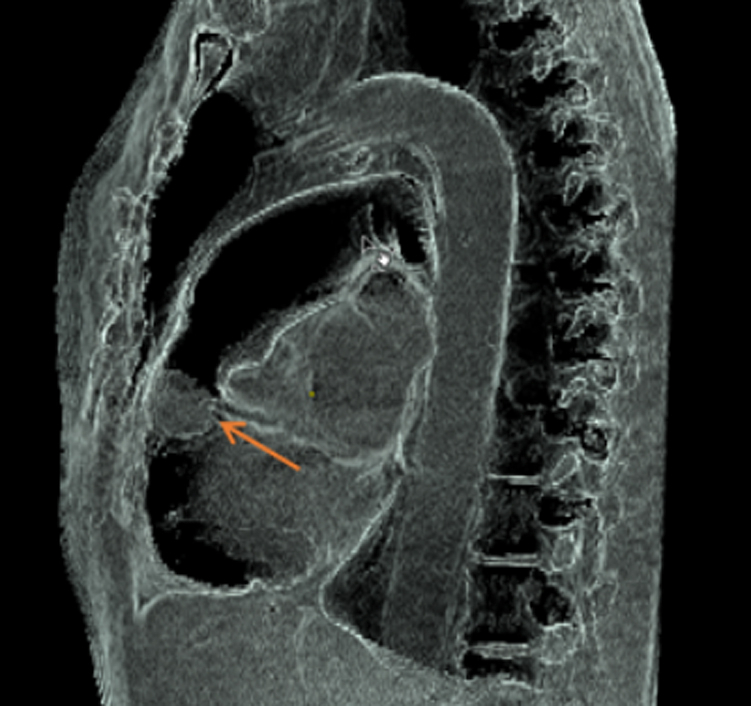
Three-dimensional reconstruction of an IgG4-related cardiac mass of the right ventricle.

**Central Message**

Cardiac mass is a rare manifestation of IgG4-related disease, a systemic disorder causing inflammatory pseudotumors. Histology is essential for the diagnosis.

## Case Presentation

**Figure 1:**
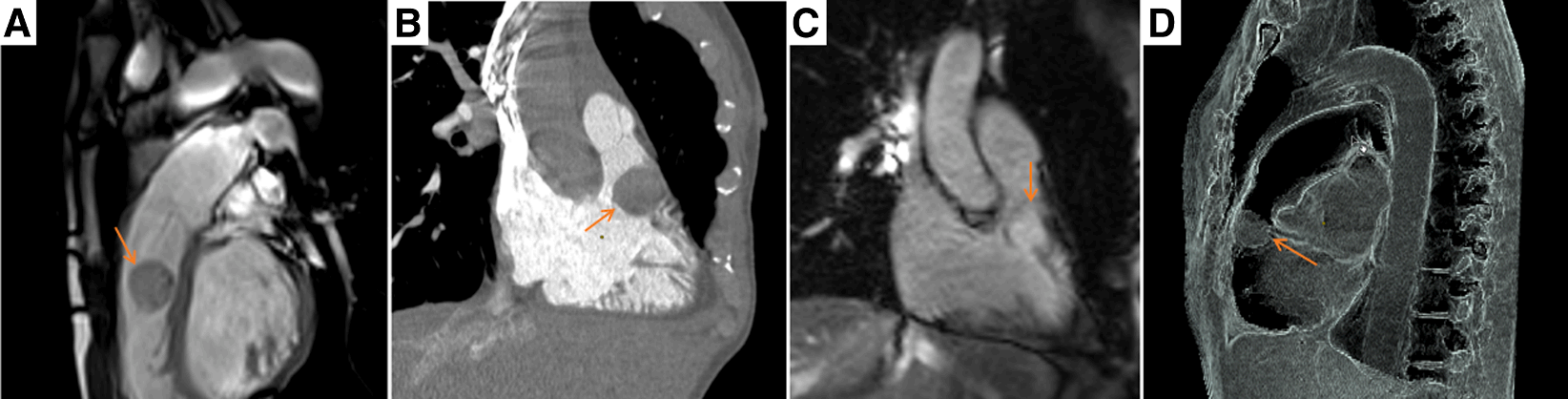
Cardiac imaging. A, Cardiac magnetic resonance imaging (*MRI*) showing a mass in the right ventricular outflow tract below the pulmonary valve (*orange arrow*). B, Computed tomography angiography showing anterior insertion in the pulmonary infundibulum. (*orange arrow*). C, Postgadolinium MRI showing homogeneous enhancement (*orange arrow*). D, Three-dimensional reconstruction of the mass (*orange arrow*).

**Figure 2:**
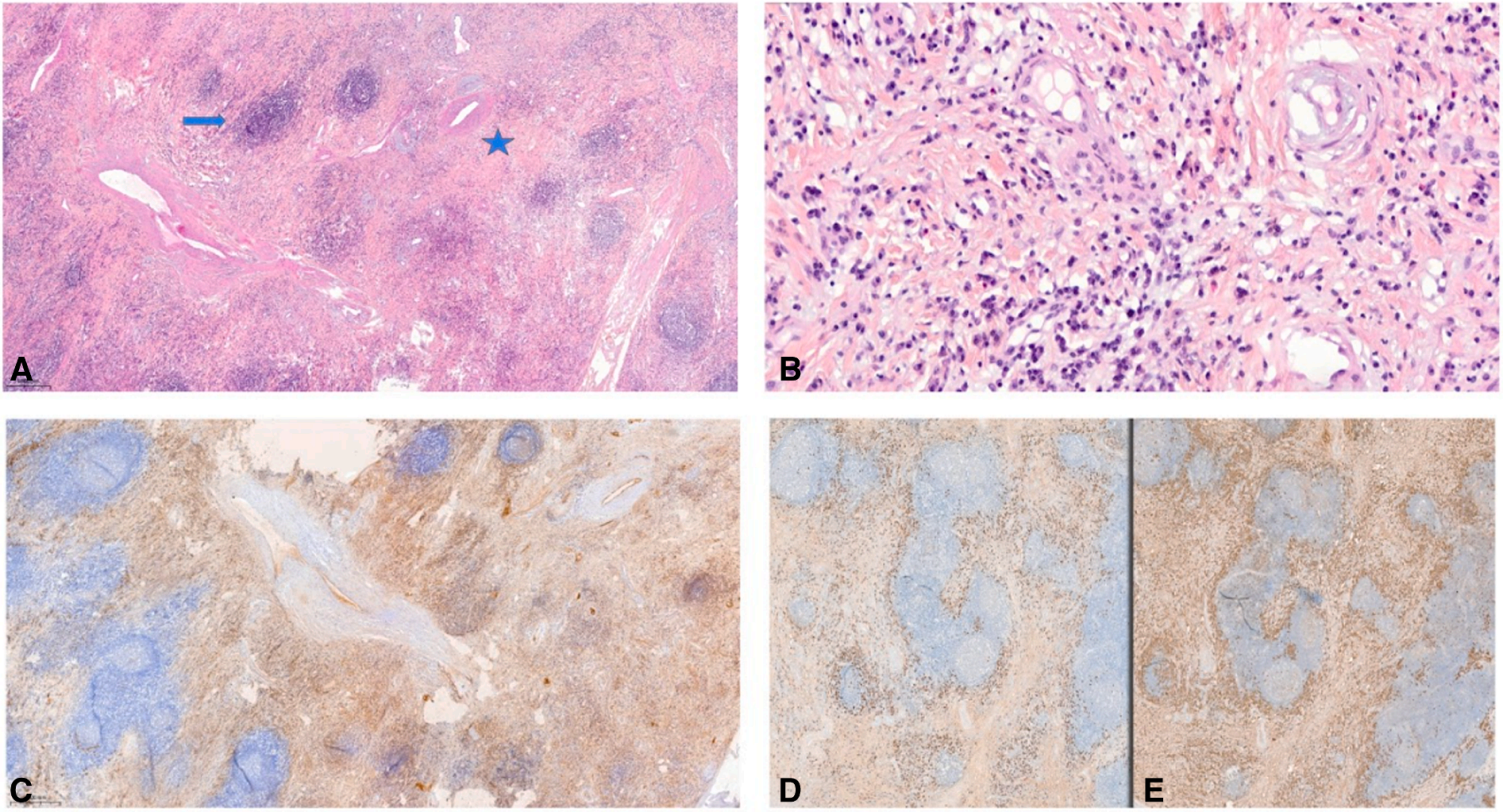
Cardiac mass biopsy. A, Fibroinflammatory infiltrate with lymphoid follicles (*blue arrow*) and storiform fibrosis (*blue star*), hematoxylin-eosin stain (*HES*) ×3. B, Storiform fibrosis with inflammatory infiltrate, HES ×30. C, Inflammatory infiltrate composed by plasma cells, marked with anti-CD138 ×3. D, Immunoglobulin G4 (*IgG4*)-related disease plasma cells infiltrate, IgG4 staining ×3. E, IgG + plasma cells infiltrate, IgG staining ×3.

A 54-year-old woman presented with palpitations of 3 months’ duration. Except for irregular pulse, the physical examination was normal. Electrocardiogram showed inferior ventricular extrasystoles. To understand these extrasystoles, an echocardiography was performed and revealed a well-delimited mass within the right ventricle (RV) measuring 24 × 21 mm that was mobile with blood flow without dilatation or RV dysfunction. Then, computed tomography (CT) and cardiac magnetic resonance imaging showed a calcified, pedunculated, nonobstructive, hyperenhanced mass in the RV outflow tract, a localization consistent with inferior ventricular extrasystoles (Figure 1 and Video 1). Invasive coronary angiography was normal. The patient underwent cardiac surgery. After a median sternotomy, cardiopulmonary bypass was established, and a right atriotomy was performed revealing an encapsulated mass within the RV outflow tract, implanted on the anterior and superior part of the interventricular septum. Total exeresis with clear margins was achieved without damaging the pulmonary valve. The septum was closed with 2 Prolene (Ethicon) *U*-stitches buttressed with polytetrafluoroethylene pledgets, and the right atrium was closed with a continuous Prolene suture. Pathological analysis revealed an inflammatory infiltrate mostly composed of immunoglobulin G4+ (IgG4+) plasma cells (Figure 2, *C* and *D*), with an IgG4/IgG ratio >50% (Figure 2, *D* and *E*), storiform fibrosis (blue star in Figure 2, *A* and *B*), and lymphoid follicle (blue arrow in Figure 2, *A*). Alcian blue staining was negative. Seric IgG, IgG4, and eosinophil tests were normal. Thoraco-abdomino-pelvic CT was normal. The patient was diagnosed with IgG4-related disease (IgG4-RD). After resection, the patient was completely asymptomatic and no specific therapy was initiated.

## Discussion

IgG4-RD is a systemic disorder with pseudotumoral organ involvement resulting from infiltration with predominant IgG4+ polyclonal plasma cells and fibrosis. Even if blood biomarkers—such as elevated serum IgG4, immunoglobulin E or eosinophilia—are evocative, histology is the cornerstone of the diagnosis.^1^ Cardiac involvement in IgG4-RD is rare, with various manifestations described. The most specific pattern is coronaritis with diffuse stenosis and aneurysms, and periarterial soft tissue thickening.^2^ Cardiac masses typically involve the aortic valve and the right cavities, without specific imaging characteristic.^3^ However the presence of IgG4-related coronaritis or paravertebral soft tissue band in chest imaging^1^ could orient toward IgG4-RD.^2^ Because the lesion was pedunculated, mobile, well-rounded, hypodense with peripheral calcifications on CT scan, and homogeneously enhanced after gadolinium injection, with intermediate T1 and T2 signal on magnetic resonance imaging, we initially suspected myxoma.^4^ Given the embolic risk of myxoma, total exeresis was decided, even if needle biopsy would have been possible to diagnose IgG4-RD. Staging workup for IgG4-RD systemic involvement was performed with a thoraco-abdomino-pelvic CT. Positron emission tomography would have been an excellent option^1^ but was unavailable. Because IgG4-RD shows excellent response to glucocorticoids and B-cell-depleting agents, medical management could have been discussed here. However, in our case as in most others, patients did not receive systemic therapy because the cardiac mass was frequently the sole manifestation of IgG4-RD, and could not have been anticipated before surgery.^3^

## Conclusions

Cardiac mass is a rare manifestation of IgG4-RD. Association with other specific lesions is suggestive of IgG4-RD but the diagnosis relies on histology. Treatment depends on systemic involvement and cardiac consequences of the mass.

## Conflict of Interest Statement

The authors reported no conflicts of interest.

The *Journal* policy requires editors and reviewers to disclose conflicts of interest and to decline handling or reviewing manuscripts for which they may have a conflict of interest. The editors and reviewers of this article reported no conflicts of interest.

## Supplementary Data

Video 1 Sequential illustration of balanced steady-state free precession sequence with mobile mass in the right ventricular outflow tract, late gadolinium enhancement showing hyperenhancement, isosignal in T1 and T2 of the mass, and computed tomography pulmonary angiography with hypodense calcified mass in the right ventricular outflow tract. Video available at: https://www.jtcvs.org/article/S2666-2736(26)00241-X/fulltext.
